# Corrigendum to: EGFR mediates LPA‐induced proteolytic enzyme expression and ovarian cancer invasion: Inhibition by resveratrol

**DOI:** 10.1002/1878-0261.12495

**Published:** 2019-05-08

The original publication by Jeong *et al*. (2012) contained inadvertent duplications in the cell invasion assay images presented in Figs 1D and 4D. The authors have corrected this by providing the original whole filter images for Figs 1 and 4 , and the revised figures and legends are included here. All authors agree to this corrigendum.

The authors apologize for any inconvenience caused.

**Figure 1:**
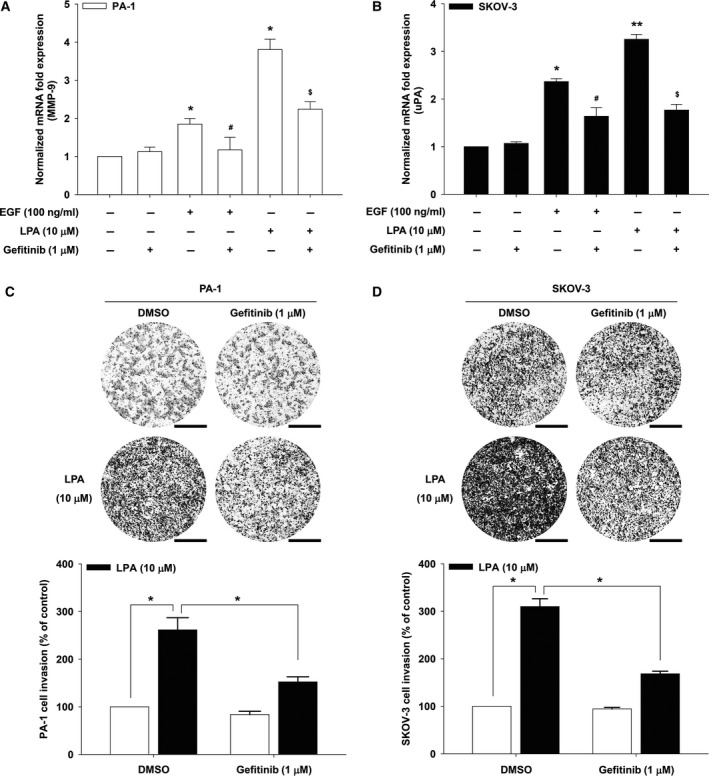
EGFR is important for proteolytic enzyme expression and ovarian cancer invasion. The serum‐starved PA‐1 (A) and SKOV‐3 (B) cells were pretreated with gefitinib for 1 h and then stimulated with LPA for 24 h. mRNA expression of MMP‐9 and uPA by quantitative RT‐PCR (error bars, ± SD *P < 0.01 and **P < 0.001 vs control, #*P* < 0.05 vs EGF treatment only, and $*P* < 0.05 vs LPA treatment only). The serum‐starved PA‐1 (C) and SKOV‐3 (D) cells were pretreated with gefitinib for 1 h, and *in vitro* invasion was analyzed by utilizing modified Boyden chamber with Matrigel‐coated polycarbonate filters against LPA (error bars, ± *SD* *P < 0.01). The values (compared with DMSO control) obtained were calculated using the number of invaded cells. Invaded cells were counted with imagej (NIH) software (image type: 16 bit, auto threshold) analyzing particles [size (pixel^2^): 100‐infinity, circularity: 0.00–1.00, exclude holes] from three filters. Scale bar, 500 µm. All experiments were repeated three times.

**Figure 4:**
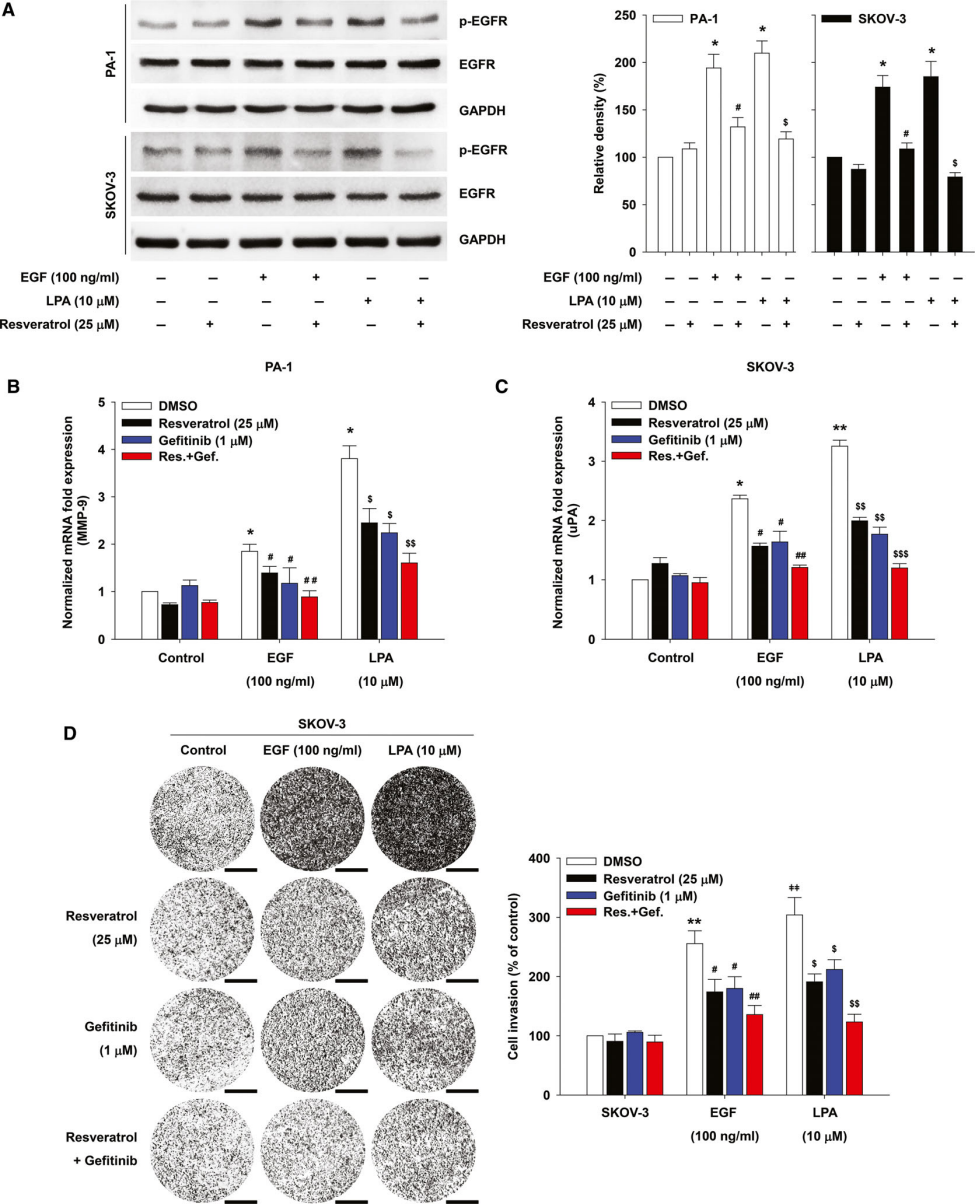
Resveratrol inhibits LPA‐induced EGFR phosphorylation and ovarian cancer cell invasion. (A) The serum‐starved cells were pretreated with resveratrol for 1 h and then stimulated with EGF or LPA for 5 min. Densitometric analysis is representative of three independent experiments. The histograms show the ratio between active and total EGFR protein levels (error bars, ± *SD* *P < 0.05 vs control, #*P* < 0.05 vs EGF treatment only, and $*P* < 0.05 vs LPA treatment only). The serum‐starved PA‐1 (B) and SKOV‐3 (C) cells were pretreated with indicated agents for 1 h and then stimulated with EGF and LPA for 24 h. mRNA expression of MMP‐9 (B) and uPA (C) by quantitative RT‐PCR (error bars, ± *SD* *P < 0.01 and **P < 0.001 vs control, #*P* < 0.05 and ##*P* < 0.01 vs EGF treatment only, and $*P* < 0.05, $$*P* < 0.01, and $$$*P* < 0.001 vs LPA treatment only). (D) The serum‐starved SKOV‐3 cells were pretreated with indicated agents for 1 h, and *in vitro* invasion was analyzed by utilizing modified Boyden chamber with Matrigel‐coated polycarbonate filters against EGF or LPA (error bars, ± *SD* *P < 0.01 vs control, #*P* < 0.05 and ##*P* < 0.01 vs EGF treatment only, and $*P* < 0.05 and $$*P* < 0.01 vs LPA treatment only). The values (compared with DMSO control) obtained were calculated using the number of invaded cells. Invaded cells were counted with imagej (NIH) software (image type: 16 bit, auto threshold) analyzing particles [size (pixel^2^): 100‐infinity, circularity: 0.00–1.00, exclude holes] from three filters. Scale bar, 500 µm. All experiments were repeated three times.

Reference

Jeong
KJ
, 
Cho
KH
, 
Panupinthu
N
, 
Kim
H
, 
Kang
J
, 
Park
CG
, 
Mills
GB
 and 
Lee
HY
 (2012) EGFR mediates LPA‐induced proteolytic enzyme expression and ovarian cancer invasion: Inhibition by resveratrol. Mol Oncol
7, 121–129.2312754710.1016/j.molonc.2012.10.001PMC5528397
